# Intracranial compliance in type 2 diabetes mellitus and its relationship with the cardiovascular autonomic nervous control

**DOI:** 10.1590/1414-431X2022e12150

**Published:** 2022-09-12

**Authors:** G.A.M. Galdino, S.C.G. Moura-Tonello, S.N. Linares, J.C. Milan-Mattos, D.L. Spavieri, S.M. Oliveira, A. Porta, T. Beltrame, A.M. Catai

**Affiliations:** 1 Laboratório de Fisioterapia Cardiovascular, Departamento de Fisioterapia, Universidade Federal de São Carlos, São Carlos, SP, Brasil; 2 Divisão de Ciência de Dados, brain4care, São Carlos, SP, Brasil; 3 Instituto de Física de São Carlos, Universidade de São Paulo, São Carlos, SP, Brasil; 4 Department of Biomedical Sciences for Health, University of Milan, Milan, Italy; 5 Department of Cardiothoracic, Vascular Anesthesia and Intensive Care, IRCCS Policlinico San Donato, Milan, Italy; 6 Samsung R&D Institute Brazil (SRBR), Campinas, SP, Brasil

**Keywords:** Cardiovascular modulation, Active postural change, Cerebrovascular circulation, Metabolic disease

## Abstract

The intracranial compliance in type 2 diabetes mellitus (T2DM) patients and the association with cardiovascular autonomic control have not been fully elucidated. The aim of this study was to assess intracranial compliance using the noninvasive intracranial pressure (niICP) and the monitoring of waveform peaks (P1, P2, and P3) and the relationship with cardiovascular autonomic control in T2DM patients. Thirty-two men aged 40-60 years without cardiovascular autonomic neuropathy (CAN) were studied: T2DMG (n=16) and control group CG (n=16). The niICP was evaluated by a noninvasive extracranial sensor placed on the scalp. Cardiovascular autonomic control was evaluated by indices of the baroreflex sensitivity (BRS), from temporal series of R-R intervals of electrocardiogram and systolic arterial pressure, during supine and orthostatic positions. The participants remained in the supine position for 15 min and then 15 min more in orthostatism. T2DMG presented a decrease of the P2/P1 ratio during the orthostatic position (P<0.001). There was a negative moderate correlation between the P2 peak with cardiovascular coupling (K^2^_HP-SAP_LF) in supine (r=-0.612, P=0.011) and orthostatic (r=-0.568, P=0.020) positions in T2DMG. We concluded that T2DM patients without CAN and cardiovascular complications presented intracranial compliance similar to healthy subjects. Despite preserved intracranial adjustments, T2DM patients had a response of greater magnitude in orthostatism. In addition, the decoupling between the heart period and blood pressure signal oscillations in low frequency appeared to be related to the worsening of intracranial compliance due to the increased P2 peak.

## Introduction

Diabetes mellitus (DM) is a chronic metabolic disease and the prevalence and incidence have been increasing worldwide (1). DM is associated with obesity, physical inactivity, inadequate diet, and metabolic syndrome (2) and affects young people and adults. The pathological process of DM damages organs such as the heart, vessels, and peripheral and autonomic nerves due to its close association with the vascular system and autonomic nervous system (ANS) (3).

The ANS comprises the sympathetic and parasympathetic nervous systems that are controlled by the sympathovagal balance (4). The imbalance is associated with the appearance of complications such as cardiovascular autonomic neuropathy (CAN), which leads to a worse prognosis and higher mortality in T2DM (5). Methods such as heart rate variability (HRV) and baroreflex sensitivity (BRS) have been widely used as they accurately assess cardiovascular autonomic control (6,7). This is possible considering stress and conditions that stimulate the sympathetic response, such as active postural changes (8).

ANS dysfunction may appear in the early stages of T2DM even in the absence of CAN (9,10), and it is speculated that cardiac vagal impairment precedes sympathetic impairment (10). There is a reduction in cardiac vagal modulation indices, decreased baroreflex sensitivity, and sympathovagal imbalance that impair blood pressure and heart rate adaptation in T2DM patients (11).

In addition, there is a significant association between ischemic stroke and decreased HRV, as well as overactivation of sympathetic activity in T2DM patients (12). Sympathovagal imbalance leads to dysregulation of cerebral circulation (5,12), although it is not entirely clear whether intracranial compliance is associated with cardiovascular autonomic control (13) in T2DM. Intracranial compliance can be analyzed through intracranial pressure waveform morphology, which is the relationship between variations in volume and pressure of the intracranial content (14). The clinical interpretation of intracranial compliance is related to the capacity of the intracranial compartment to accommodate to volume changes. In this sense, changes in intracranial pressure waveform morphology may indicate an impairment in intracranial compliance, which can help predict brain function deterioration (14).

We hypothesized that T2DM patients without CAN will present a decrease in intracranial compliance compared with a control group. Furthermore, this altered compliance could be associated with impaired cardiovascular autonomic modulation. Therefore, this study aimed to evaluate the intracranial compliance in T2DM without CAN and its relationship with cardiovascular autonomic control.

## Material and Methods

### Study design and patient selection

This was a cross-sectional study developed in the Cardiovascular Physical Therapy Laboratory (LFCV) of the Federal University of São Carlos (Brazil). Participants were recruited from the LFCV, Center of Medical Specialties of São Carlos database, and electronic media. The screening period was from March 2017 to November 2018. The study was performed according to the principles of the Declaration of Helsinki and was approved by the Ethics Committee of the Federal University of São Carlos. All participants signed a written consent form with procedure explanations prior to participation.

Men aged between 40 to 60 years with T2DM and without CAN and apparently healthy subjects matched for age were recruited. Women were not included to avoid the influence of hormonal factors in the cardiovascular autonomic control. Moreover, only T2DM without CAN were included to verify only the early influence of T2DM on cardiovascular autonomic control. The inclusion criteria were: T2DM diagnosis of at least 6 months performed by a physician; body mass index (BMI) ≤35 kg/m^2^; non-smokers, non-alcoholics, and non-narcotics users; and without orthopedic alterations, stroke history, acute myocardial infarction, and respiratory diseases. The control group included subjects without diagnosis of cardiovascular, cerebrovascular, pulmonary, or metabolic diseases.

The exclusion criteria were: CAN, sensorimotor alterations, and other diabetic neuropathies diagnosis for the T2DM group; use of betablockers, antidepressants, anxiolytics; and acute inflammatory disease, peripheral arterial obstructive disease, and electrocardiographic abnormalities at rest and during exercise. All subjects were matched for age, weight, stature, and BMI.

### Experimental procedures

After database screening, the subjects were invited to perform the assessments at the LFCV. Initially, demographic and general data including age, weight, stature, BMI, duration of diabetes, comorbidities, and healthy conditions were collected. Sensorimotor sensitivity [(evaluated by a 10-g Semmes-Weinstein monofilament (Sorri-Bauru, Brazil)], ankle brachial index, and cardiovascular autonomic reflex tests (CARTs) were evaluated. The CARTs proposed by Ewing et al. (15) are the gold standard for CAN diagnosis (16) and were used to identify this condition in diabetes patients and exclude those with two or more abnormal tests. The heart rate (HR) responses to the deep breathing test (normal if ≥1.21), Valsalva maneuver (normal if ≥1.21), and the 30:15 ratio (normal if ≥1.04) were evaluated. The systolic arterial pressure (SAP) response was also assessed during postural challenge (abnormal response: reduction at ≥30 mmHg) (15). CARTs followed the recommended standardized methods (17) and the HR interpretation was based on normal age-related values. For other diabetic neuropathies, a clinical anamnesis was performed. Moreover, participants were asked to restrain from physical exercise and coffee consumption before the tests. Participants had a light meal 2 h before the CART battery.

Afterwards, each participant completed the assessment process in three more stages. First, a physician and physical therapist at the LFCV conducted a clinical exercise test on a cycle ergometer to assess aerobic capacity and detect possible electrocardiographic abnormalities. Second, body composition was evaluated by dual x-ray absorptiometry to analyze the total lean mass, total fat mass, and fat percentage. Finally, fasting glucose, glycated hemoglobin, C-reactive protein, and lipid profile were collected before the experimental protocol by a specialized laboratory. The experiment consisted of cardiovascular autonomic control evaluation by means of HRV, systolic arterial pressure variability (SAPV), BRS, and intracranial compliance at rest and in orthostatic position measured through noninvasive intracranial pressure (niICP) monitoring.

### Cardiovascular autonomic control

Two days before the experimental protocol, participants were familiarized with the equipment and procedures. All patients were instructed to have a regular night's sleep, avoid caffeinated or alcoholic beverages, and refrain from strenuous physical exercise at least 24 h before and on the day of the experiment. The experiment was carried out in the morning in a silent room at the LFCV with controlled temperature (22-23°C) and relative air humidity (40-60%) (18). Before starting data collection, the participants remained at rest for 10 min to stabilize the signals (18). After that, they remained in a supine position for 15 min and then changed to an orthostatic position, where they remained for 15 min. Participants were instructed to breathe spontaneously and not to move or talk during the experiment.

The R-R intervals (RRi) were captured by electrocardiogram (ECG) from lead II (BioAmp FE132, ADInstruments, Australia). In addition, respiratory movements were captured by a piezoelectric belt attached to the patient (Marazza, Italy) to obtain the respiratory rate during testing. Arterial pressure (AP) was continuously measured, beat-to-beat, by a photoplethysmography (Finometer PRO; Finapress Medical System, The Netherlands) on the middle finger of the left hand. The RRi, AP, and respiratory movement signals were sampled at 1 kHz using a commercial acquisition device (PowerLab 8/35; ADInstruments).

The assessment of cardiovascular autonomic control consisted of time domain, spectral, and cross-spectral analysis. After detecting the QRS complex on the ECG signal, the R-wave apex was identified using a parabolic interpolation. The heart period (HP) was estimated by calculating the temporal distance between two consecutive parabolic vertices. SAP was considered as the maximum value of the AP wave for each heartbeat (19). A stable sequence of 256 beats (for SAP and HP) was chosen in the supine and orthostatic positions, and if isolated ectopic beats were present they were linearly interpolated (19). From these 256 beat samples, the HP mean (μHP), HP variance (σ^2^HP), SAP mean (μSAP), and SAP variance (σ^2^SAP) were calculated in the time domain. The BRS in time domain was estimated by searching for an HP and SAP sequence that presented four consecutive beats that increased (ascending sequence) or decreased (descending sequence) simultaneously in the same direction (20). In addition, the sequence selection criteria (19) were: total variation of HP >5 ms, total variation of SBP >1 mmHg, and the correlation coefficient between the HP and SAP variations higher than 0.85. The slope of the regression line between the series (HP and SAP) was calculated. The mean angles of regression were named αSEQ and the percentage was defined as %SEQ (21).

HP and SAP variability parameters were assessed according to the autoregressive model (19). The spectral components were decomposed into low frequency (LF, from 0.04 to 0.15 Hz) and high frequency (HF, from >0.15 to 0.4 Hz) reported in absolute (ms^2^ and mmHg^2^) and normalized units (n.u.) (6). The BRS was evaluated by cross-spectral analysis using a bivariate autoregressive parametric approach (19). The time series relations between SAP and HP were represented as coherence, phase, and transfer function gain. The coherence function shows the degree of association between the HP and SAP (K^2^_HP-SAP_) variabilities (7,22). Its values vary from 0 to 1, where values closer to 1 represent a better coupling between signals. The phase represents the time relation between the series, i.e., the delay between the change in the SAP signal resulted from the heartbeat (Ph_HP-SAP_) and was evaluated in the HF (Ph_HP-SAP_HF) and LF (Ph_HP-SAP_LF) bands. The transfer function gain (αTF) (7,23) represents how much the output signal (i.e., HP) changes for a given input signal change (i.e., SAP) and was calculated as described previously (7). The αTF (22) was analyzed in the HF and LF (αTFHF and αTFLF, respectively) bands.

### Intracranial compliance recording and analysis

The intracranial compliance was evaluated using the niICP monitoring device Brain4Care^®^ (BcMM 1500, brain4care, Brazil). This method and device were previously validated (24,25). The niICP monitoring was captured continuously using an extracranial sensor placed on the left hemisphere in the electroencephalography C5 point (located above the ear, in the center of the temporoparietal transition, two inches perpendicularly above the entrance to the external auditory canal in the coronal plane). All volunteers rested in supine position with their heads resting on a pillow approximately 10 centimeters high, positioned at 11 degrees on a rigid stretcher with the headboard at 0 degrees. The sensor (strain gauge) allows measuring the volumetric variations of the skullcap and shows the shape of the pulse wave and converts it into graphic results.

The graphics of the niICP monitoring represents three characteristic peaks: P1 is the *percussion wave* that represents the blood pressure exerted under the choroid plexus and is related to cerebral blood flow. P2 is the *tidal wave*, it is variable and indicates the brain tissue compliance due to reflection of the P1 wave. P3 is known as the *dicrotic wave* due to the closure of the aortic valve (26). These peaks determine the intracranial compliance through its relationship with cerebral blood volume. Intracranial compliance considers the ability of the brain and the cranial cavity to adapt to (or tolerate) variations in volume and pressure of the intracranial content (14). The response to the intracranial content cause micro-oscillations in the walls of the skullcap leading to cranial expansion. In normal conditions, the peaks are in P1>P2>P3 order and the P2/P1 ratio is <1, representing adequate compliance. However, when the P2 peak (peak related to the intracranial compliance) is greater than the P1 peak, due to sustained ICP increase, it can indicate pathological morphology (27). The increased P2 peak can lead to a marked decrease in intracranial compliance assuming the P2/P1 ratio >1 (28).

**Figure 1:**
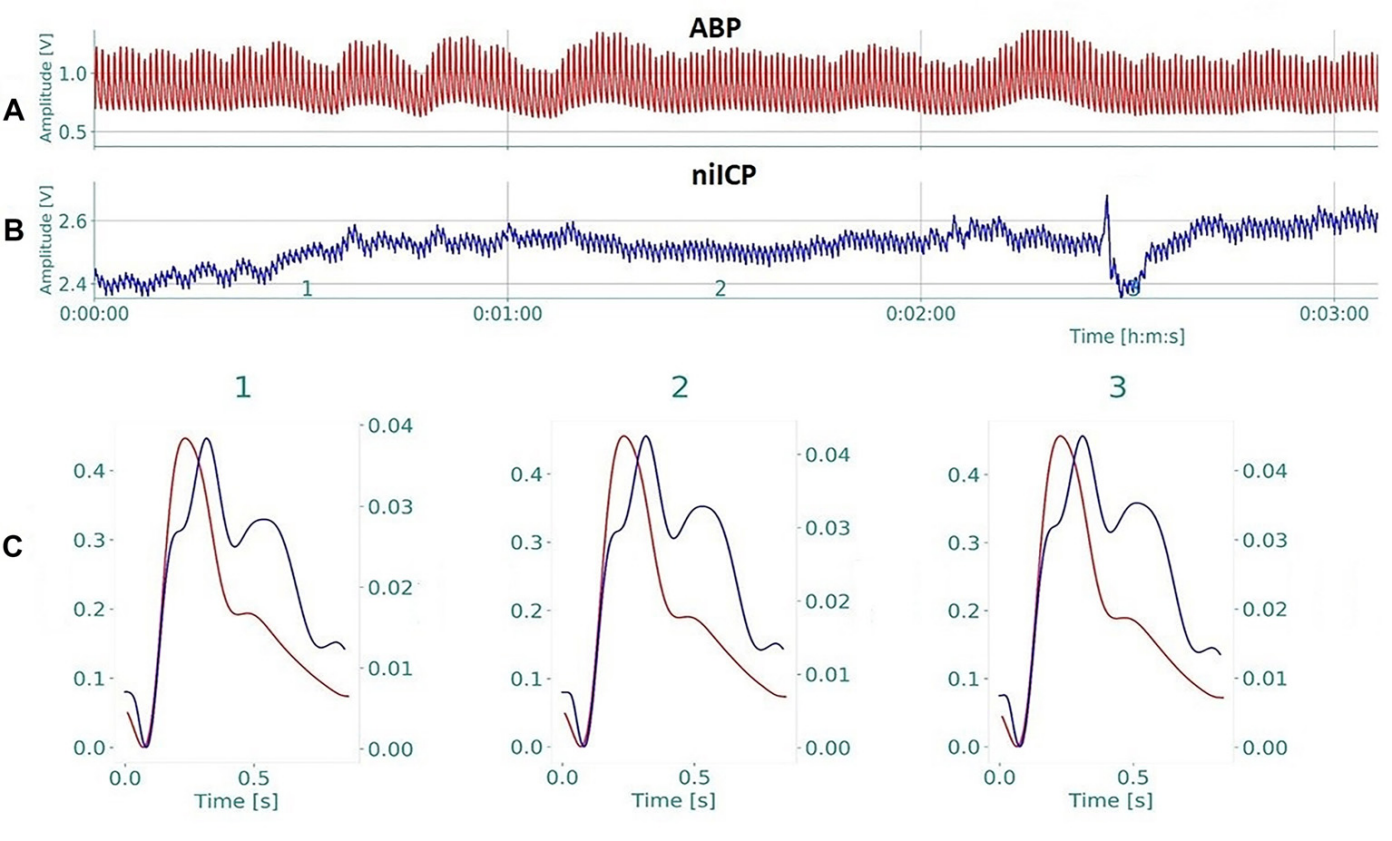
Noninvasive intracranial pressure waveform identification. **A**, acquisition of the arterial blood pressure (ABP) wave over time; **B**, noninvasive intracranial pressure (niICP) waveform; **C**, joint analysis of blood pressure (in dark red) and noninvasive intracranial pressure (in dark blue) waveforms during 3 minutes of data collection in type 2 diabetes mellitus patients.

The niICP monitoring and sensor calibration were conducted according to manufacturer specifications. The waveform was visualized in real time and the signal was coupled to a PowerLab 8/35 data acquisition board (AD Instruments, Australia) sampled at 1 kHz. The waveforms data were analyzed by a blind evaluator using Braincare Analytics software (version 5d1665, brain4care, Brazil). P1 and P2 peaks were determined from the mean pulse of the time-paired AP wave. The P1 peak was estimated from the AP waveform maximum amplitude and the P2 peak from the start of diastole (dichrotic node), also from the AP waveform. P2 was defined as the point corresponding to the mean time between P1 and the beginning of diastole (Figure 1). The 95% confidence interval (α=0.05) is represented as a gray region around the midline of the niICP. One minute of the niICP signals in supine position and standing with the most stable data, i.e., with the lowest artifact interference, was chosen for further analysis.

### Statistical analysis

Data distribution normality was verified by the Shapiro-Wilk test. Descriptive data are reported as the mean, standard deviation, median (interquartile range), and percentage (%). Differences between T2DM and control groups for physical characteristics, laboratory analysis, ankle brachial index, and medications were compared using Student's *t*-test for normal distribution data and the Mann-Whitney test for non-normal distribution data.

Two-way mixed ANOVA with Student-Newman-Keuls *post hoc* was used to compare niICP waveforms between groups (T2DMG *vs* control group) within the same condition (supine *vs* orthostatic), between conditions within the same group (factor A: group; factor B: position) and their interactions. HRV, SAPV, and BRS indexes were also compared between groups within the same condition.

Spearman's correlation was performed to assess the relationship between intracranial compliance and cardiovascular autonomic control. In addition, we subdivided the T2DM group to assess how diagnosis time and glycated hemoglobin levels influenced the relationship of cardiovascular autonomic control and intracranial compliance. The correlations were classified as low (0.26 to 0.49); moderate (0.50 to 0.69), or high (0.70 to 0.89).

Linear multiple regression was also performed to explain the interference of family risk factors on niICP waveform in the control group because some subjects presented the P2/P1 ratio >1 in the supine and orthostatic positions. The following variables were considered: P1, P2, P2/P1 ratio in supine and orthostatic positions, and the presence of diabetes, hypertension, myocardial infarction, obesity, and stroke in the family history of each subject. Statistical analysis and figures were performed using Sigma Plot software version 11.0 (Systat Software, Inc., USA), and the significance level was fixed at 5%.

## Results

### Demographic and clinical characteristics

**Figure 2:**
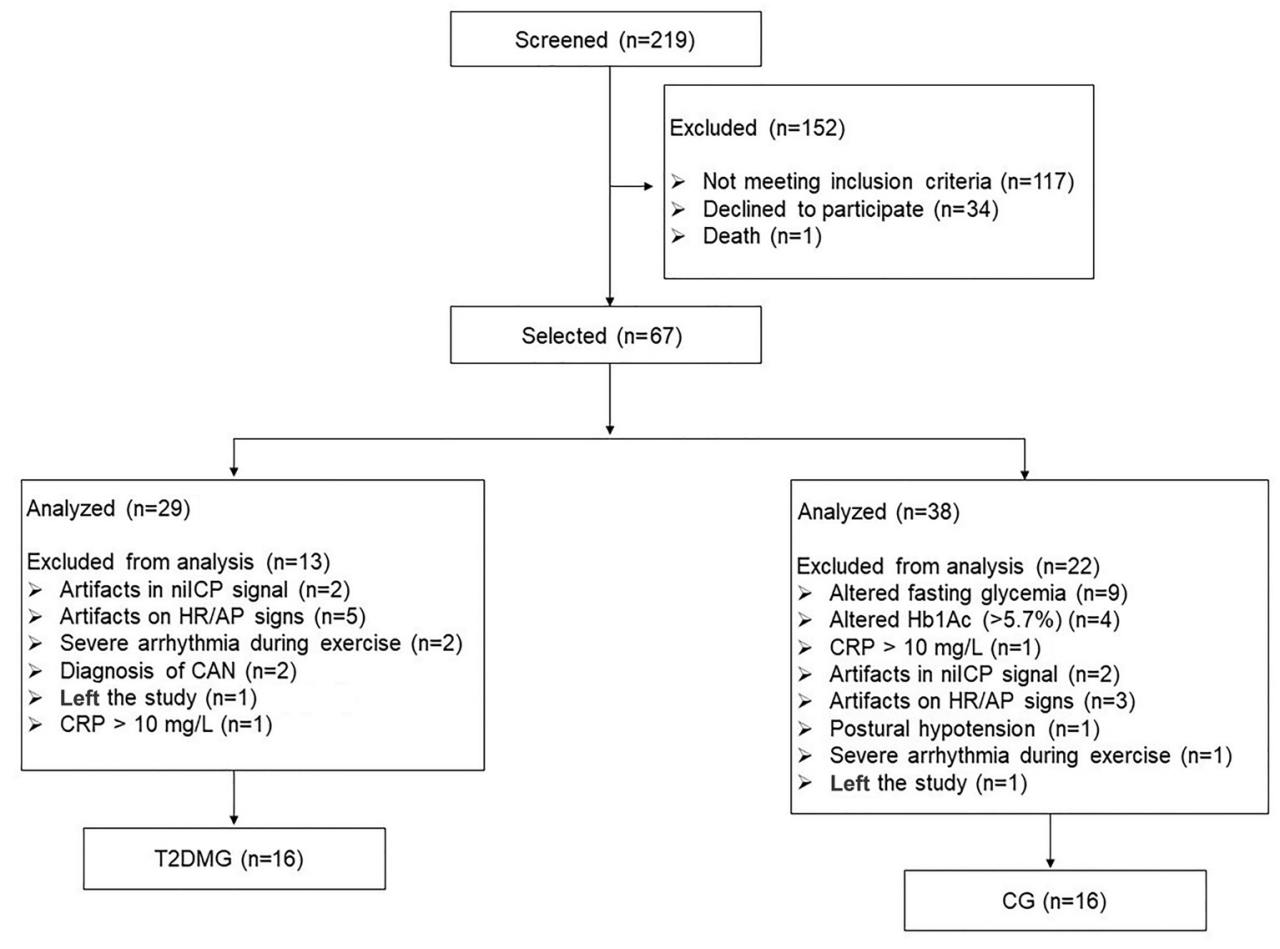
Flow chart of participants in the study. niICP: noninvasive intracranial pressure; HR: heart rate; AP: arterial pressure; CAN: cardiovascular autonomic neuropathy; CRP: C-reactive protein; HbA1c: glycated hemoglobin; T2DMG: type 2 diabetes mellitus group; CG: control group.

Initially, 219 subjects were screened for the study of which 152 were excluded because they did not meet the inclusion criteria. Then, 67 subjects were selected for the study: 29 patients with T2DM and 38 apparently healthy individuals. From this sample, 13 patients from the T2DM group and 22 subjects from the control group were excluded due to clinical and signal alterations. Thus, the final sample size consisted of 32 men: T2DM group without CAN (T2DMG; n=16) and control group (CG; n=16) (Figure 2).

The T2DMG had normal mean CART values and significantly increased HR at rest and higher waist-to-hip ratio compared to CG. T2DMG presented higher fasting blood glucose, glycated hemoglobin, and triglycerides than CG (Table 1).

**Table 1:** Anthropometric, clinical, and laboratory characteristics of study subjects.

Characteristics	CG (n=16)	T2DMG (n=16)	P value
Age (years)	49±4	53±6	0.058
Anthropometric data			
Body mass (kg)	82.84±10.76	86.27±10.49	0.369
Height (m)	1.74±0.06	1.73±0.06	0.633
BMI (kg/m^2^)	27.28±2.67	28.62±2.29	0.139
Clinical data			
Time of diabetes (years)	-	10±6	-
HR rest (beats/min)	59±9	69±12	0.014*
SAP (mmHg)	129±18	128±14	0.809
DAP (mmHg)	72 (65-80)	71 (67-74)	0.559
Brachial ankle index (right)	1.15±0.14	1.10±0.12	0.318
Brachial ankle index (left)	1.12±0.15	1.07±0.11	0.311
Total lean mass (kg)	57.58±7.32	59.51±7.03	0.469
Total fat mass (kg)	21.48±5.01	23.95±5.00	0.187
A/G ratio	1.11±0.13	1.24±0.14	0.018*
% Fat (%)	26.06±3.36	27.71±3.42	0.193
Cardiovascular risk factors			
Hypertension (%)	-	3 (17.64)	-
CARTs			
Deep breathing (E/I ratio)	-	1.257±0.133	-
Valsalva maneuver	-	2.228±0.629	-
30:15 ratio	-	1.093±0.093	-
SAP response to standing (mmHg)	-	6.222±7.138	-
Laboratory exams			
Fasting plasma glucose (mg/dL)	94 (89-98)	168 (127-194)	<0.001*
HbA1c (%)	5.3 (5.1-5.5)	8.1 (6.7-8.9)	<0.001*
Insulin (μU/mL)	8.3 (6.4-10.5)	10.4 (6.8-31.0)	0.085
Total cholesterol (mg/dL)	207±27	213±49	0.656
HDL-cholesterol (mg/dL)	44±9	40±8	0.226
LDL-cholesterol (mg/dL)	136±22	130±34	0.574
VLDL-cholesterol (mg/dL)	21 (18-26)	33 (23-40)	0.079
Triglycerides (mg/dL)	106 (94-132)	170 (131-223)	0.018*
CRP (mg/L)	1.60 (0.66-2.19)	1.07 (0.73-2.86)	0.806
Medications (n, %)			
Metformin	-	5 (31.25)	-
Metformin + insulin	-	1 (6.25)	-
Metformin + sulfonylurea	-	3 (18.75)	-
Metformin + sulfonylurea + insulin	-	2 (12.50)	-
Metformin + insulin + DPP-4 enzyme inhibitor	-	1 (6.25)	-
Insulin	-	2 (12.50)	-
Inhibitor of ACE	-	2 (12.50)	-
Hypolipidemic	-	2 (12.50)	-

Data are reported as means±SD, median (interquartile range), and number (percentage). CG: control group; T2DMG: type 2 diabetes mellitus group; BMI: body mass index; HR: heart rate; SAP: systolic arterial pressure; DAP: diastolic arterial pressure; A/G: android-to-gynoid ratio; CARTs: cardiovascular autonomic reflex tests; E/I ratio: expiration/inspiration; HbA1c: glycated hemoglobin; HDL: high-density lipoproteins; LDL: low-density lipoproteins; VLDL: very-low-density lipoproteins; CRP: C-reactive protein; DPP-4: dipeptidyl peptidase 4; SGLT2: sodium-glucose co-transporter 2; ACE: angiotensin converting enzyme. *P<0.05 (*t*-test or Mann-Whitney test).

### Cardiovascular autonomic control

Both groups showed a reduction in the μHP in orthostatism and T2DMG presented lower values than the CG in both positions (significantly lower in supine position). The σ^2^HP significantly decreased in the standing position in both groups. Still in the time domain, the baroreflex sensitivity (αSEQ) was significantly reduced in both groups after adopting the orthostatic position. The percentage of baroreflex sensitivity gain (%SEQ) increased during orthostatism in both T2DMG and CG, however no difference was found between the groups (Table 2).

**Table 2:** Characterization of cardiovascular autonomic control of the study subjects.

	CG (n=16)		T2DMG (n=16)		P value		
	Supine	Orthostatic	Supine	Orthostatic	Group	Position	Interaction
μHP (ms)	1029.99 (143.67)§	849.57 (115.10)*	888.52 (153.24)	734.62 (127.90)*	0.009	<0.001	0.329
σ^2^HP (ms^2^)	2179.30 (2022.75)	1481.31 (1487.84)*	1181.53 (1299.77)	563.74 (438.05)*	0.055	<0.001	0.802
μSAP (mmHg)	129 (18)	128 (22)	128 (14)	127 (13)	0.842	0.600	0.867
σ^2^SAP (mmHg^2^)	27 (13)	37 (19)	33 (23)	36 (18)	0.522	0.186	0.452
αSEQ (ms/mmHg)	10.61 (4.53)	6.54 (3.12)*	8.20 (4.61)	4.29 (2.01)*	0.094	<0.001	0.945
%SEQ	5.20 (4.90)	10.24 (7.17)*	4.74 (4.22)	8.90 (4.62)*	0.543	<0.001	0.552
HF_HP_ (ms^2^)	529.63 (619.76)§	154.80 (176.34)*	226.56 (258.16)	44.53 (52.11)	0.017	<0.001	0.029
LF_HP_ (n.u.)	60.26 (18.22)	78.71 (13.51)*	63.32 (17.01)	82.93 (12.83)*	0.421	<0.001	0.857
LF_SAP_ (mmHg^2^)	9.70 (5.00)	25.12 (16.25)*	11.05 (14.70)	26.81 (18.13)*	0.684	<0.001	0.957
αLF (ms/mmHg)	8.39 (4.40)	5.07 (2.69)*	6.86 (3.71)	3.42 (1.02)*	0.118	<0.001	0.920
αHF (ms/mmHg)	14.02 (7.52)§	6.62 (3.95)*	6.82 (4.31)	4.18 (3.18)*	0.004	<0.001	0.431
K^2^_HP-SAP_LF	0.83 (0.07)	0.90 (0.06)*	0.80 (0.15)	0.90 (0.06)*	0.621	<0.001	0.431
Ph_HP-SAP_LF (rad)	-0.92 (0.38)	-1.13 (0.35)	-1.20 (0.43)	-1.18 (0.23)	0.140	0.146	0.085
K^2^_HP-SAP_HF	0.86 (0.14)	0.90 (0.09)	0.89 (0.05)	0.85 (0.12)	0.802	0.861	0.133
Ph_HP-SAP_HF (rad)	-0.18 (0.41)	-0.33 (0.67)	-0.43 (0.60)	-0.38 (0.72)	0.441	0.607	0.297
αTFLF (ms/mmHg)	13.01 (6.11)§	6.91 (3.91)*	8.86 (5.37)	4.18 (1.98)*	0.026	<0.001	0.336
αTFHF (ms/mmHg)	16.09 (8.99)§	6.99 (3.82)*	9.87 (5.25)	5.19 (2.74)*	0.029	<0.001	0.957

Data are reported as means (SD). T2DMG: type 2 diabetes mellitus group; CG: control group; μ: average; HP: heart period; σ^2^: variance; SAP: systolic arterial pressure; αSEQ: baroreflex sequence; %SEQ: sequence percentage; HF: high frequency; LF: low frequency; ms: milliseconds; n.u.: normalized units; αLF and αHF: baroreflex sensitivity in the LF and HF bands; K^2^_HP-SAP_LF and K^2^_HP-SAP_HF: HP-SAP coherence in LF and HF bands; Ph_HP-SAP_LF and Ph_HP-SAP_HF: HP-SAP phase in LF and HF bands; rad: radian; αTFHF and αTFLF: transfer function gain in LF and HF bands. §P<0.05 between groups; *P<0.05 between positions (two-way mixed ANOVA).

In the frequency domain, T2DMG presented a significantly lower value of the HF_HP_ component in absolute units compared with the CG in the supine position. There was a statistical difference between the positions only in the CG with a reduction of the value in the standing position. Also, there was an interaction between groups and positions. The LF_HP_ component (nu) was significantly increased in both T2DMG and CG in the standing position. There was no difference between groups for the LF_SAP_ component (mmHg^2^), but both showed an increase in the orthostatic position. The BRS gain by the spectral method in the LF component (αLF) significantly decreased in orthostatic position in both groups. In the HF band (αHF), the CG showed a significant increase compared with the T2DMG in the supine position, and both showed a decrease when adopting the orthostatic position.

In cross-spectral analysis, K^2^_HP-SAP_LF significantly increased in the orthostatic position in both groups. There was no difference between groups and positions for the phases in LF and HF, and in coherence (K^2^_HP-SAP_) in the HF band. T2DMG showed significantly lower values of αTFLF and αTFHF compared to CG in the supine position. Both groups showed a significant reduction in αTFLF and αTFHF when adopting the orthostatic position.

### Intracranial compliance

**Figure 3:**
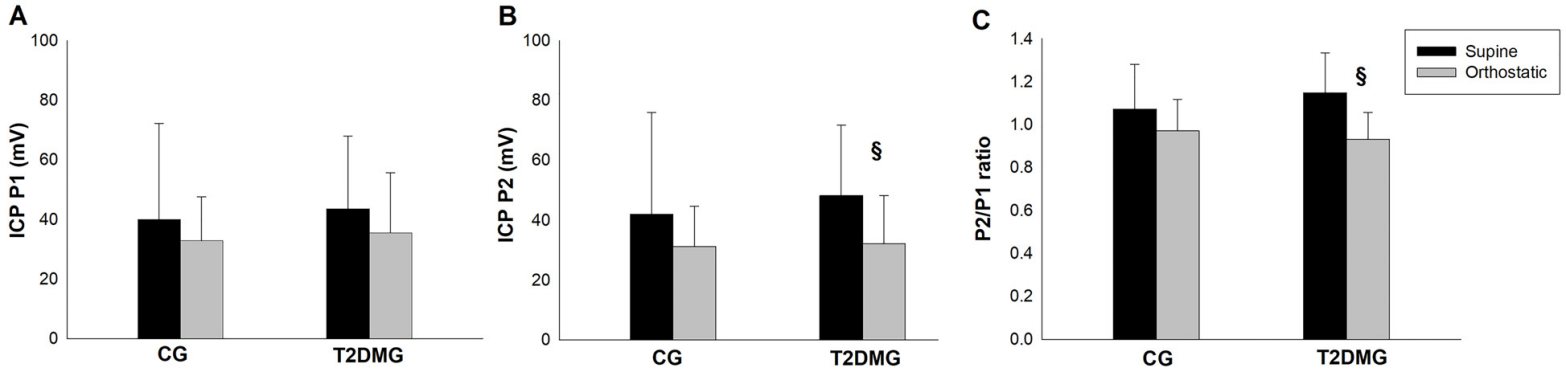
Characterization of noninvasive intracranial pressure (ICP) in type 2 diabetes mellitus patients (T2DMG) compared with controls (CG). **A**, noninvasive ICP P1 wave; **B**, noninvasive ICP P2 wave; **C**, intracranial compliance waveform. Data are reported as means±SD. ^§^P<0.05 supine *vs* orthostatic positions (two-way mixed ANOVA).

Regarding the analysis of the variable P1 (Figure 3A), no differences were observed between CG and T2DMG (P=0.609) and between positions (P=0.202). Likewise, no differences were found for variable P2 (Figure 3B) between groups (P=0.557). However, T2DMG presented a significant reduction (P=0.021) in the orthostatic position compared to the supine position. For the P2/P1 ratio, no differences were found between groups (P=0.669); however, there was a significant decrease (P2/P1 ratio <1; P<0.001) during the orthostatic position compared to the supine position in the T2DMG (Figure 3C).

When analyzing the variation magnitude (orthostatism-supine) of the niICP indexes, only the T2DMG presented a variation of the P2 peak (ΔP2) (P=0.034) and P2/P1 ratio (Δ P2/P1) (P<0.001) during active postural change.

### Association of intracranial compliance with cardiovascular autonomic control

**Figure 4:**
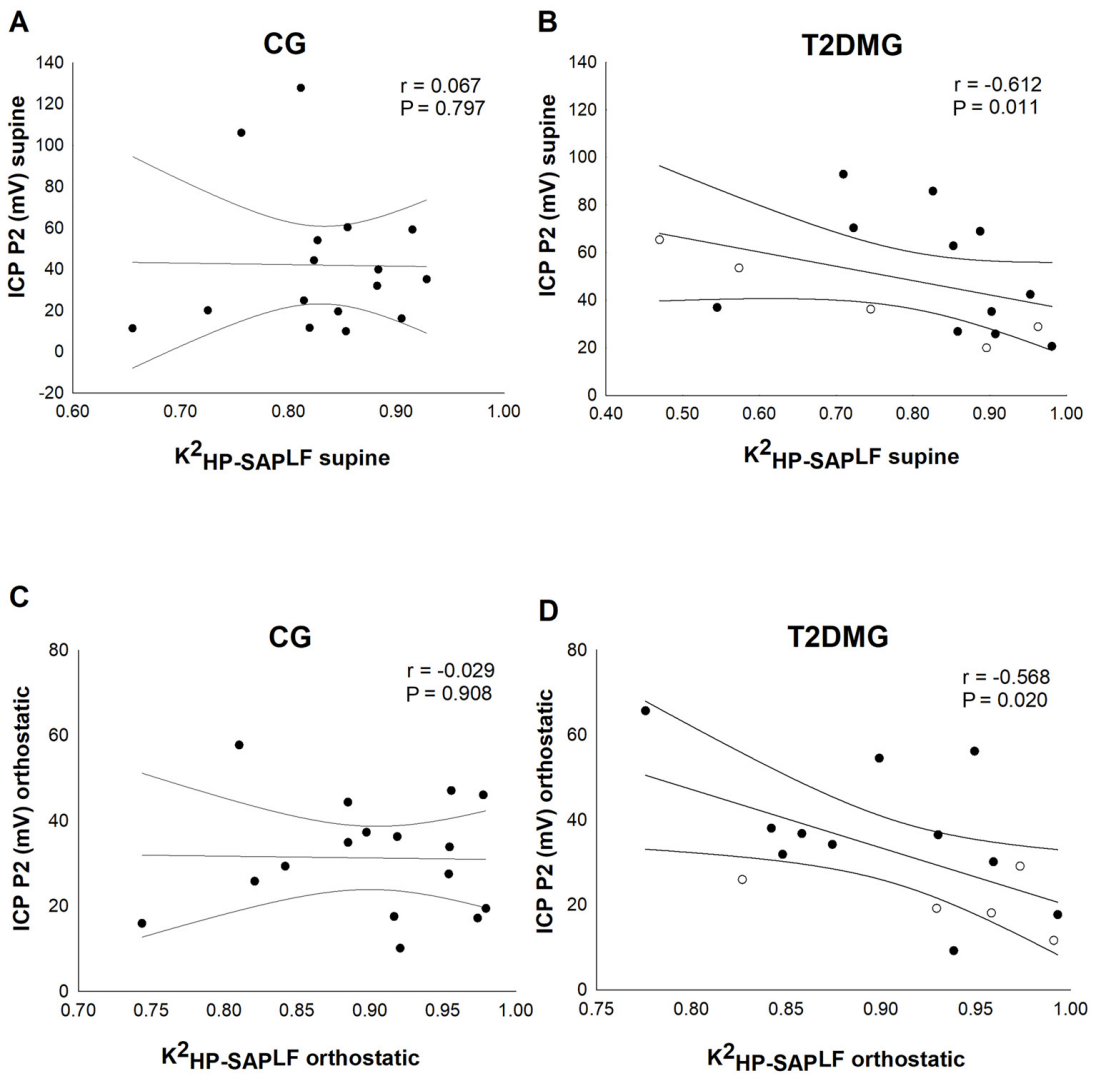
Correlation between noninvasive intracranial pressure (ICP) and cardiovascular coupling (K^2^_HP-SAP_LF) in type 2 diabetes mellitus patients (T2DMG) compared with control (CG). **A** and **B**, correlation between the noninvasive ICP P2 wave in the supine position. **C** and **D**, noninvasive ICP P2 wave in the orthostatic position with the cardiovascular coupling. Open circles: glycated hemoglobin less than 7%. Closed circles: glycated hemoglobin more than 7%. Spearman’s correlation was used for analyses.

There was a negative and moderate association between the K^2^_HP-SAP_LF and P2 peak in supine (Figure 4A and B) and orthostatic positions (Figure 4C and D) in T2DMG, showing reduced coupling between HP and SAP signs (i.e., reduced coherence) with an increase in the P2 wave amplitude.

**Figure 5:**
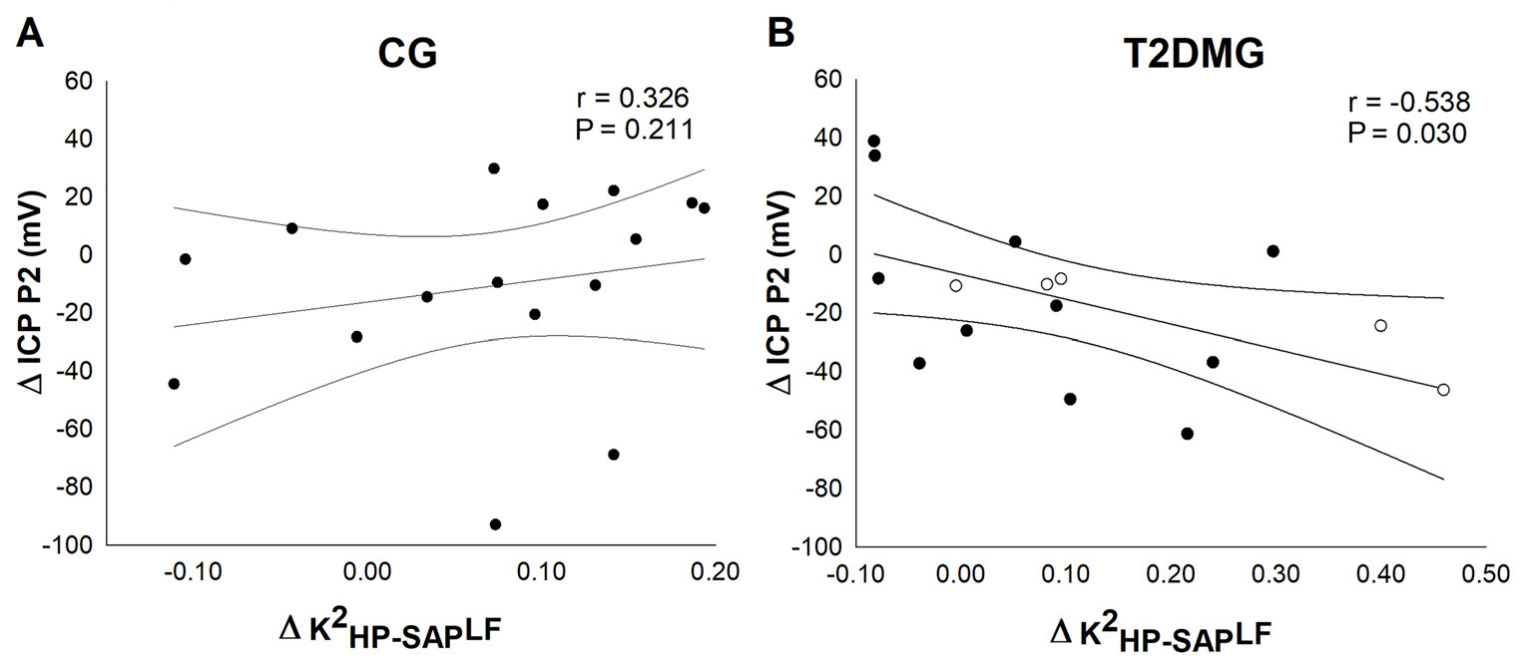
Correlation between noninvasive intracranial pressure (ICP) and variation of cardiovascular coupling (K^2^_HP-SAP_LF). **A** and **B**, noninvasive ICP P2 wave and cardiovascular coupling variation in type 2 diabetes mellitus patients (T2DMG) compared with controls (CG). Open circles: glycated hemoglobin less than 7%. Closed circles: glycated hemoglobin more than 7%. The Spearman’s correlation was used for analyses.

There was a negative and moderate correlation between the variation (orthostatic-supine) of P2 peak and the coherence in the LF band in T2DMG (Figure 5B). This suggested that variations (reduction) in cardiovascular coupling were related to an increase in the P2 wave amplitude in T2DM patients.

### Impact of disease duration and glycated hemoglobin levels

When we subdivided the T2DM in controlled (HbA1c <7%, n=5) and uncontrolled (HbA1c >7%, n=11) glycated hemoglobin (29), a moderate and negative correlation (r=-0.655, P=0.026) was identified between the P2 peak and the K^2^_HP-SAP_LF for the uncontrolled T2DM in supine position. Furthermore, subdividing the T2DM group (n=16) by diagnosis time (time <5 years, n=5 and time >5 years, n=11), a moderate and negative correlation (r=-0.627, P=0.035 and r=-0.645, P=0.029) was identified between the P2 peak and the K^2^_HP-SAP_LF for those diagnosed more than 5 years ago in supine and standing positions, respectively.

### Influence of family history on intracranial compliance

Linear multiple regression was performed to evaluate the influence of family risk factors on intracranial pressure variables in the CG. The presence of a family history of diabetes mellitus and myocardial infarction (P=0.044 and P=0.047, respectively) were able to predict the P2/P1 ratio >1 in the supine position in the CG. In addition, subjects with a family history of stroke had an increase in P1 (P=0.001) and P2 peaks (P=0.007) in the supine position.

## Discussion

The main findings of this study were: 1) intracranial compliance of T2DM was similar to control subjects in both supine and orthostatic positions; 2) active postural change induced a significant decrease in P2 waveform and P2/P1 ratio only in T2DMG; 3) the reduced cardiovascular coupling [(coherence between HP and SAP in the LF band (in supine position and orthostatism)] was associated with increased P2 peak (associated to intracranial compliance) in T2DM patients.

Although the initial hypothesis of the study was statistically rejected, to the best of our knowledge, this was the first study to investigate the behavior of intracranial compliance and the correlation with the ANS in T2DM patients without CAN. Thus, our study may contribute to a better understanding of the integration between the cardiovascular ANS and the cerebrovascular system in T2DM during a simple physiological stimulus such as active postural challenge.

In our study, the intracranial compliance noninvasively evaluated by P1 and P2 peaks and P2/P1 ratio did not differ between T2DM and healthy subjects in both positions. Physiological brain response to postural change is documented in the literature (30). Supine is the main position for ICP monitoring due to the decrease in cerebral venous return by the internal jugular vein, accumulation of fluid, increased volume with consequently ICP increase (30). This could justify the increase in P1 and P2 peaks in the supine position in both groups. A slight increase in intracranial volume affects pressure in the brain. As a response, pressure rises to adapt to volume variations. However, the ability to adapt is not linear, therefore in these cases, compliance decreases and assumes P2/P1 ratio values greater than 1 (14). This explains why both T2DM and healthy subjects presented an expected response of the intracranial compliance in the supine position.

During the orthostatic position, T2DM presented a decrease in the P2 peak and P2/P1 ratio compared to the supine position. In the standing position, there is a significant decrease in blood flow in the internal jugular vein compensating hydrostatic pressure, reducing mean arterial pressure, and redirecting cerebrospinal fluid to alternative routes such as the vertebral venous plexus, leading to a drop in ICP (31). A recent study with Doppler showed that individuals with T2DM have reduced cerebral blood flow and altered cerebral hemodynamics (32). Cerebrovascular reactivity is also altered in these patients (32). We speculate that, despite the good ability to regulate ICP in orthostatism, the greater drop in P2 peak and P2/P1 values of the T2DM group after active postural change may be due to changes in flow regulatory factors. These changes facilitate intracranial volume and pressure reduction. Based on our results, we can infer that T2DM patients without CAN and microvascular or macrovascular complications do not present changes in intracranial compliance compared with healthy controls.

Studies have provided evidence of the stressor effect of active postural maneuver in the cardiovascular system, leading to an acute cardiac vagal withdrawal (8) and vascular sympathetic activation (10). Our data corroborated the results of de Moura-Tonello et al. (10), who observed lower cardiac vagal modulation and impaired baroreflex control of the cardiac period in the supine position and increased cardiac and vascular sympathetic modulation in the orthostatic position in patients with T2DM without CAN (10). This sympathetic increase acutely contributes to BRS reduction in healthy subjects (7) and T2DM patients (10). Previous studies with T2DM (9,10) showed that the disease *per se* in the early stages is able to modify the cardiac vagal modulation at rest, evaluated by cardiovascular variability indexes. The ability of the T2DM group to maintain baroreflex and sympathetic control of vessels in orthostatic position suggests that the absence of CAN or other neuropathies maintains the sympathetic responsiveness in T2DM patients. Although no difference was found between the groups for the coherence in the LF and HF band, αSEQ, and BRS gain in the LF and HF band after orthostatic stress, as in another study with the same population (10), there was a difference between the positions within each group, which was expected (10,33). Therefore, it can be suggested that our group of patients had a preserved cardiac baroreflex response to changes in systolic arterial pressure considering sympathetic stressors compared with the control group.

In the present study, the K^2^_HP-SAP_LF showed a significant, moderate, and negative correlation with P2 waveform in supine position and orthostatism. The coherence index is more commonly used to verify the reliability of the BRS calculation, as changes in HP must accompany variations in SAP and be sufficiently coherent and significant (22). Thus, few studies have reported the use of coherence only to characterize the control of cardiac baroreflex (33). However, the association of coherence with intracranial compliance found in our study may indicate that a loss of cardiovascular control capacity, during the cardiac period to respond to SBP variations in different positions, is due to cardiac vagal impairment and greater influence of sympathetic modulation (33). Less coherence may mean less involvement of the baroreflex in the regulation of interactions between HP and SAP (22). Thus, our results suggested that early changes in the coupling of cardiac period oscillations and systolic artery pressure with the influence of sympathetic modulation can contribute to worsening cerebral adjustments in T2DM patients without CAN. However, further investigations are necessary to better explore the correlations found between coherence and intracranial pressure waveforms morphology.

Studies have demonstrated that instability in arterial pressure regulation due to lower BRS in T2DM is associated with increased risk of stroke due to factors that involve the loss of cerebral autoregulation, an increase in the cerebrovascular sympathetic tone leading to an increase in vascular resistance (34). Thus, the ANS may influence brain responses. Considering that cardiovascular adjustment depends on the ANS, which requires cardiac vagal modulation, cardiac/vascular sympathetic modulation, or baroreflex sensitivity activation (4), there may be a relationship between ICP and BRS by the sympathetic output (35). Guild et al. (36) evaluated intracranial pressure and renal nerve sympathetic activity in animals to identify a new pathway for the management of ICP and AP through the sympathetic nervous system. The authors found that a progressive increase in ICP reflects an increase in AP and HR. One explanation would be that neurons in the rostral ventral lateral medulla (RVLM) are barosensitive and elicit a pressure response when stimulated (37).

The analysis of the impact of controlled glycated hemoglobin or time of diagnosis in T2DM suggested that patients with worse glycemic control and disease duration of more than 5 years may have less cardiovascular coupling and a higher P2 peak. Poor glycemic control, that is, high levels of glycated hemoglobin, can predict the development of microvascular and macrovascular complications (38). Furthermore, complications such as chronic kidney disease, cardiovascular disease, and autonomic and peripheral neuropathies have been described in patients with prolonged disease (39). Thus, we suggest that poor glycemic control and the long-term disease may directly impact blood vessels, blood pressure, and autonomic nerves such as ANS. Therefore, complications induced by glycemic control and disease duration may reflect an impairment of intracranial compliance, leading to an increase in the P2 peak amplitude.

The evaluation of family risk factors within the control group was necessary to explain the P2/P1 ratio >1 in ten healthy subjects in supine position and eight subjects in orthostatic position. Our results suggested that family history of diabetes mellitus, acute myocardial infarction, and stroke may negatively influence the intracranial compliance of apparently healthy subjects. However, further genetic analysis studies are necessary to understand the influence of heredity on changes in intracranial pressure in apparently healthy subjects (40). Thus, early investigation of risk factors may reduce/delay the onset of cerebrovascular complications in subjects who have not been diagnosed with T2DM but have a family history of the disease.

Therefore, our study suggested that T2DM patients without CAN have adequate intracranial compliance in the supine and orthostatic positions. We can infer that compliance is not affected in the early stages of the disease. Moreover, the significant association between the coherence of HP-SAP and ICP waveform morphology in the present study suggested that an adequate cardiovascular coupling strength plays a role in the behavior of the reflected wave (P2). However, more studies are necessary to better elucidate the association of baroreflex sensitivity using other indexes and methodologies with intracranial compliance in T2DM patients with and without CAN. Assessing the influence of CAN is essential to comprehend whether disease severity and cardiovascular autonomic dysfunction worsens intracranial compliance.

Our study has limitations that must be addressed. The small number of participants is a limiting factor for the generalization of findings to the T2DM population. In addition, the device used for noninvasive monitoring of ICP does not provide values in millimeters of mercury, only in millivolts. However, previous research by the group showed that the analysis of waveform morphology in millivolts is similar to that collected by the invasive technique (24). Moreover, neurological images such as magnetic resonance imaging or conventional transcranial Doppler could not be evaluated before the experimental protocol. These technologies would be useful to assess brain structure and directly measure blood flow in cerebral arteries to better understand cerebral hemodynamics in patients with T2DM without CAN. Despite limitations, the study provides relevant information about the relationship between intracranial compliance and cardiovascular coupling, and encourages further investigations on autonomic and baroreflex variables in the behavior of cerebral compliance in T2DM patients.

In the present study, T2DM patients without CAN and cardiovascular complications were shown to present intracranial compliance similar to healthy subjects. Despite preserved intracranial adjustments, these patients had a greater magnitude of response in orthostatism. Moreover, the reduction in the coupling of cardiovascular oscillations in T2DM patients was associated with impairment of intracranial compliance even in the absence of neuropathies.
